# Design and validation of a qualitative interview for the study of the role of physical activity in urban public spaces in the social inclusion of immigrant women (Spanish version)

**DOI:** 10.3389/fspor.2026.1717266

**Published:** 2026-03-02

**Authors:** Ana Blanco-Ayala, Angela Magnanini, Daniel Medina-Rebollo, Jesús Fernández-Gavira

**Affiliations:** 1 Department of Physical Education and Sports, University of Seville, Seville, Spain; 2 Department of Movement, Human and Health Sciences, Foro Italico University of Rome, Rome, Italy; 3 School of Humanities, Education and Sport, Universidad CEU Fernando III, CEU Universities, Bormujos, Seville, Spain

**Keywords:** immigrant women, social inclusion, content validity, physical activity, public space, qualitative research, sport sciences

## Abstract

**Introduction/objective:**

This study aimed to validate a semi-structured qualitative interview developed to examine how physical activity in urban public spaces contributes to processes of social inclusion and acculturation among immigrant women;

**Methods:**

A mixed-method validation process was carried out with the participation of five experts in social sciences, sport studies, and qualitative research. The evaluation combined quantitative measures - using Aiken's V and the Content Validity Coefficient (CVC) - with qualitative assessments. Experts also provided detailed feedback regarding the clarity, coherence, cultural sensitivity, and thematic balance of the proposed items;

**Results:**

The quantitative analysis showed high levels of content validity, with mean values of Aiken's V = 0.89 and CVC = 0.88, confirming strong agreement on the relevance and clarity of the items. Qualitative feedback highlighted the need for adjustments in wording, reorganization of sections, and the inclusion of items addressing family context, migration trajectories, and gender-related perceptions. Following these recommendations, the interview was expanded from 44 to 54 items, structured across ten thematic sections;

**Discussion/conclusion:**

The resulting instrument offers a useful and flexible methodological tool to investigate the experiences of immigrant women in relation to physical activity and participation in urban public spaces, while incorporating dimensions of social inclusion, acculturation, and gender. Its capacity for adaptation makes it valuable for future studies related to these topics across diverse contexts.

## 1 Introduction

The sustained growth of the immigrant population in Spain over the past decades has raised new challenges in terms of integration, social cohesion, and the design of inclusive policies. In particular, the significant increase in the number of immigrant women (1) requires specific attention from social, urban, and health research, since their experiences of acculturation and social inclusion are shaped by multiple and intersecting structural inequalities. Several studies have shown that migrant women often face additional barriers during their adaptation processes, stemming from the intersection of factors such as gender, class, ethnicity, and legal status (2–4).

These inequalities are not limited to individual trajectories but are also spatially expressed through the use and appropriation of urban public spaces, particularly those associated with physical activity. Access to and participation in physical activity in public environments are influenced by gendered norms, socioeconomic constraints, and experiences of exclusion, making these spaces key sites where social inequalities are reproduced and contested (5–7).

Within this context, the AFISAMI (Physical Activity, Social Inclusion, and Acculturation of Immigrant Women) project provides the empirical framework for the present study and is aimed at understanding how physical activity in urban public spaces may contribute to processes of social inclusion and the strengthening of social networks among immigrant women in situations of vulnerability. Findings from earlier phases of the project have highlighted the relevance of urban physical activity as a context for social interaction and acculturation processes among immigrant women (8).

Despite this growing body of evidence, there remains a need for methodological tools capable of capturing immigrant women's lived experiences of physical activity, social inclusion, and acculturation within urban public spaces. Such tools must be sensitive to power relations, gendered dynamics, and intersectional inequalities, in line with critical qualitative and constructivist approaches in social and sport research (9–11).

### 1.1 Theoretical framework

This study is grounded in a theoretical framework that integrates three interrelated dimensions: physical activity, social inclusion processes, and acculturation among immigrant women, with particular attention to their experiences in urban public spaces.

From a migration and social integration perspective, immigrant women often face multidimensional challenges that hinder full participation in social life. These challenges are shaped by the intersection of gender, migratory status, socioeconomic position, ethnicity, and cultural norms, generating structural inequalities in access to resources, social networks, and public spaces (2, 3, 12). Social inclusion is therefore conceptualized not merely as individual participation, but as a relational and structural process involving access, recognition, and the capacity to appropriate social and urban environments.

Urban public spaces play a central role in these dynamics. Beyond their physical characteristics, they function as social arenas where inclusion and exclusion are negotiated through everyday practices, norms, and power relations. Factors such as management, accessibility, safety, and symbolic meanings condition who can use these spaces, how, and under what circumstances. For immigrant women, urban public spaces may simultaneously represent opportunities for social interaction and physical activity, as well as sites of surveillance, insecurity, or cultural tension (13, 14).

Within this context, physical activity practiced in urban public spaces can operate as a mediating mechanism between acculturation and social inclusion. Acculturation is understood as a dynamic and bidirectional process through which individuals negotiate cultural practices, identities, and social relations within the host society. Participation in physical activity and sport has been shown to facilitate social interaction, language acquisition, and the development of social capital, while also contributing to well-being and a sense of belonging (4, 6, 15). However, access to these potential benefits is uneven, as cultural expectations, gender norms, discrimination, and caregiving responsibilities may restrict immigrant women's opportunities to engage in physical activity in public spaces.

Adopting an intersectional and critical perspective allows physical activity to be conceptualized not merely as a health-related behavior, but as a socially situated practice embedded in power relations and urban dynamics. This perspective is essential for understanding how immigrant women experience, negotiate, and attribute meaning to physical activity within public spaces, and how these experiences shape broader processes of social inclusion and acculturation (9, 10).

This theoretical framework provides the conceptual basis for the design and validation of the semi-structured interview guide, ensuring that the instrument captures the complexity of immigrant women's lived experiences at the intersection of physical activity, urban space, and social integration.

## 2 Study objective

The objective of this study was to carry out a systematic content validation process, from an intersectional perspective, of a semi-structured interview guide designed to examine processes of social inclusion and acculturation among immigrant women, with particular attention to their participation in physical activities within the city's urban public spaces.

## 3 Methodology

The present study employed a descriptive methodological design based on a combined quantitative and qualitative validation strategy to assess the content validity of a semi-structured interview guide through expert judgment. This approach, widely recognized in the specialized literature, was designed to ensure the relevance, clarity, and coherence of the instrument, while maintaining sensitivity to the social and cultural contexts of immigrant women's experiences.

The validation of the instrument was carried out in three phases, following the methodological model proposed by Sánchez-Uzcátegui (28), which integrates a review of the state of the art, instrument design, and both quantitative and qualitative validation through expert judgment.

### 3.1 Phase one: review of the literature

The first stage of the methodological process consisted of a systematic literature review, conducted in line with the Preferred Reporting Items for Systematic Reviews and Meta-Analyses (PRISMA) guidelines (16), the results of which were published in Blanco-Ayala et al. (8). This review aimed to establish the theoretical and conceptual frameworks that would guide the design of the interview guide, as well as to define the key dimensions to be addressed. The analysis focused on studies examining the relationship between physical activity, social inclusion processes, and acculturation among immigrant women, as well as research exploring the role of sport as a tool for integration and the creation of community bonds (4, 6, 15).

The review identified four critical dimensions for understanding the phenomenon: (1) the reciprocal influence between sport/physical activity participation and acculturation, (2) the presence of cultural and gender-based barriers, (3) the impact of structural and socioeconomic factors, and (4) the effects on the psychosocial well-being of immigrant women. These dimensions informed both the content and the structure of the instrument, underscoring the need for an approach that articulates bodily practices with the social and spatial conditions shaping migratory trajectories (3, 13, 14, 17).

Additionally, a critical and intersectional perspective (2, 12, 18) was incorporated to capture the complexity of women's experiences in urban contexts marked by inequalities of gender, ethnicity, class, and migratory status. This perspective was essential to ensure the relevance and sensitivity of the guide to the concrete realities of participants.

In parallel, a methodological review was conducted to identify suitable procedures for the validation of qualitative instruments through expert judgment. Foundational works by Aiken (19), Hernández-Nieto (20), and Ventura-León (21) were considered, along with empirical studies applied in diverse fields (22–24). Notably, Sánchez-Uzcátegui's (28) work on the validation of an interview guide for Venezuelan migrant women in Spain served as a methodological reference for this study, due to its rigor and alignment with a qualitative approach informed by a critical perspective.

### 3.2 Phase two: design of the interview guide

The semi-structured interview guide was designed for immigrant women who met the following inclusion criteria: a) aged 18 years or older, b) residing in Spain for at least one year, c) participating in or expressing interest in physical activities in urban public spaces, d) representing diverse nationalities, and e) experiencing conditions of social or economic vulnerability. Situations of social or economic vulnerability were defined using functional and contextual criteria commonly applied in social and community-based research. Specifically, vulnerability was identified based on the presence of one or more of the following conditions: unstable or low-income employment, unemployment, limited access to social or institutional support resources, precarious housing conditions, caregiving responsibilities combined with limited support networks, or experiences of social exclusion related to migratory status, gender, or ethnicity. These criteria were assessed through self-reported information and contextual knowledge provided by community organizations involved in participant recruitment.

The guide was developed using both inductive and deductive approaches, integrating findings from the systematic review and theoretical frameworks on acculturation, social networks, inclusive urbanism, gender, and intersectionality. Its structure was organized into ten thematic sections, each with defined objectives and open-ended questions designed to capture personal experiences, barriers, facilitators, perceptions, and proposals for improvement. These sections were:

1. Sociodemographic information
2. Migratory and cultural background
3. Current physical activity in leisure time and sports spaces
4. Use and perception of public spaces
5. Social inclusion and support networks
6. Impact on the process of social inclusion and perceived well-being
7. Gender perceptions in physical activity
8. Public policies and recommendations
9. Focus groups
10. Final reflections and closing remarks

The wording of the guide was carefully designed to be accessible and adaptable to other languages. Technical jargon and terms that might induce bias or discomfort were avoided. In addition, grammatical, semantic, and syntactic coherence was ensured, following the recommendations of Juárez-Hernández and Tobón (25) on language quality in qualitative instruments.

Given the linguistic and cultural diversity of the target population, the interview guide was originally developed in Spanish and subsequently adapted for use with participants who did not have sufficient proficiency in the host language. When necessary, the instrument was translated into the participants' preferred language with the support of bilingual mediators or professionals experienced in intercultural contexts. Special attention was paid to preserving conceptual and semantic equivalence, rather than literal translation, in order to ensure comprehensibility and cultural appropriateness of the questions.

This phase concluded with a preliminary version of the instrument consisting of 44 questions distributed across the 10 sections, which was subsequently subjected to a content validation process, as described below.

### 3.3 Phase three: quantitative and qualitative validation of the interview guide

To ensure the content validity and methodological quality of the interview guide, a dual validation process was conducted - quantitative and qualitative - through the judgment of five experts with extensive research and professional experience in fields related to the study's focus (physical activity, social inclusion, migration, gender, and qualitative research). This phase was crucial to guarantee the relevance, clarity, and coherence of the instrument, as well as its alignment with the general and specific objectives of the project.

Although the interview guide developed in this study is qualitative in nature and semi-structured in design, the validation process followed a quantitative content validity approach. Specifically, expert judgment was used to assess the adequacy, clarity, and relevance of each item, and the degree of agreement among experts was quantified through statistical indices. In this sense, the study does not seek to validate constructs or predictive relationships, but rather to establish the content validity of a qualitative research instrument by means of quantitative indicators of expert agreement, namely Aiken's V and the Content Validity Coefficient (CVC).

This mixed methodological logic—qualitative instrument development combined with quantitative content validation—is widely accepted in methodological research, particularly in the validation of semi-structured interviews intended for qualitative inquiry.

#### 3.3.1 Selection of experts

Five experts were contacted (Table 1), meeting the following inclusion criteria: postgraduate training in related disciplines (sociology, occupational therapy, sport management, gender studies, and social sciences), experience in qualitative research, ethical commitment, and prior participation in instrument validation processes. Disciplinary, institutional, and geographical diversity were also prioritized. Exclusion criteria included: failure to respond affirmatively to the invitation or informed consent, lack of minimum qualifications and experience, and evidence of manifest bias or conflict of interest compromising objectivity in validation. All experts contacted formally accepted participation, signing the informed consent for the validation process.

**Table 1:** Profile of validators.

Validator	Profile description
1	University professor. Occupational therapist. Associate Professor, Department of Community Medicine and Rehabilitation, Umeå University (Sweden), Occupational Therapy Section.
2	University professor. Sociologist specializing in the sociology of sport. Full Professor of Sociology, University of Córdoba (Spain).
3	University professor. Occupational therapist. Lecturer, Division of Occupational Therapy, University of Cape Town (South Africa), and researcher in the Inclusive Practices Africa group.
4	University professor. Sociologist. Associate Professor, Department of Sociology, Pablo de Olavide University, Seville (Spain).
5	Social and cultural anthropologist. Manager and consultant in socio-sport projects. Director of community initiatives and president of a non-profit association (Seville, Spain).

Source: own elaboration.

#### 3.3.2 Evaluation process

Each expert received, via email, the full validation instrument, the informed consent form, and two templates: one for the quantitative evaluation of each item (on a Likert scale from 1 to 5, where 1 = Poor and 5 = Excellent), and another for qualitative assessment, with space to provide free-text comments per item and per section.

The experts reviewed all 44 items across the 10 thematic sections. The evaluation process took place between April and July 2025, through virtual meetings and asynchronous document review.

#### 3.3.3 Quantitative validation: calculation of Aiken's V and the content validity coefficient (CVC)

To determine content validity, two coefficients were calculated for each item:

Aiken's V coefficient was used to assess the degree of agreement among experts regarding the relevance of each item. The formula adapted by Penfield and Giacobbi (26) was applied, considering items valid with a V ≥ 0.70 in accordance with the methodological literature (19, 33):

$$V=\overset{}{\underset{}{\sum }}\left(\frac{r_{i}-l}{n(c-l)}\right)$$

Where:

*r* = score assigned by expert to item *i*
*l* = minimum value of the scale (1)
*c* = maximum value of the scale (5)
*n* = number of experts

The CVC was calculated following the procedure proposed by Hernández-Nieto (20), which allows for the quantitative assessment of content validity based on expert judgment. The CVC evaluates both the degree of agreement among experts and the adequacy of each item with respect to the construct or domain under study, and has been widely discussed as a methodological approach to content validity in applied research (27).

First, experts rated each item using a five-point Likert scale ranging from 1 (poor) to 5 (excellent). For each item, the arithmetic mean of the experts' scores (${\bar{x}}_{i}$) was calculated and divided by the maximum possible score of the scale (*c* = 5), yielding an initial CVC value.

The corrected CVC for each item was obtained by subtracting this error term from the initial coefficient, as recommended by Hernández-Nieto (20):

$$CVC_{i}=\frac{{\bar{x}}_{i}}{c}-Pe_{i}$$

Where:

${\bar{x}}_{i}$ = mean score of item *i*
*c* = maximum value of the scale (5)
Pe*_i_* = estimation error for item *i*

Items with a corrected CVC value equal to or greater than 0.80 were considered to have adequate content validity. This threshold was applied both at the item level and to assess the overall content validity of the instrument

To correct for potential bias associated with the number of experts, an estimation error (*Pe_i_*) was calculated as the inverse of the number of experts:

$$Pe_{i}=\frac{1}{n}$$

Where:

*n* = number of experts

All calculations were performed using Microsoft Excel 2019.

#### 3.3.4 Qualitative validation: expert observations and consensus

In addition to quantitative validation, a qualitative validation process was undertaken through a detailed analysis of the written observations provided by the five experts. Feedback was collected per item and per section, and systematized to identify convergences, divergences, and specific suggestions. This approach complemented the psychometric analysis with a critical assessment of non-quantifiable aspects such as language accessibility, conversational flow, cultural appropriateness, and thematic sensitivity of the guide.

The research team applied predefined criteria to decide on the incorporation of qualitative suggestions, prioritizing those that: (a) were agreed upon by at least two experts, (b) were supported by solid methodological or ethical reasoning, and (c) aligned with the objectives and approaches of the AFISAMI project. The procedure followed the methodological guidelines proposed by Sánchez-Uzcátegui (28) and Hernández-Nieto (20), thereby ensuring the traceability and rigor of the decisions made.

#### 3.3.5 Final consolidation

Based on the combined analysis of quantitative and qualitative results, the research team developed a revised version of the interview guide, integrating the agreed-upon modifications while preserving items deemed conceptually relevant. The final version was organized according to internal logic, discursive coherence, and semantic sensitivity. Decisions regarding wording, sequencing, and reformulation were made collectively, with the aim of maximizing the clarity, relevance, and applicability of the instrument for future qualitative fieldwork. The final validated version of the interview guide is available in the Supplementary Material.

## 4 Results

The findings from the validation process of the interview guide are presented at two levels: quantitative and qualitative.

### 4.1 Quantitative results

#### 4.1.2 Aiken's V coefficient

The analysis of Aiken's V coefficient showed that 39 out of the 44 items (88.6%) reached or exceeded the minimum acceptable threshold of 0.70, the value established in the specialized literature as the validity criterion. The overall mean value of Aiken's V was 0.89, which reflects a high degree of agreement among the experts regarding the relevance of the instrument's items.

The items with the highest ratings (V = 1.00) were 1.1, 1.2, 1.4, 1.6, 1.7, 1.8, 2.2, 4.2, 4.3, 6.1, 6.4, and 10.2. Conversely, the items with the lowest scores were 9.3 (V = 0.70), and 2.3, 3.1, 3.4, 3.6, and 7.4 (all with V = 0.75), which therefore required priority review.

#### 4.1.3 Content validity coefficient (CVC)

The corrected CVC values, calculated following Hernández-Nieto's procedure, also showed satisfactory results. The overall CVC of the instrument was 0.88, well above the minimum threshold of 0.80. At the item level, 22 items (50%) obtained a corrected CVC ≥ 0.80 and were considered valid without modification.

A total of 15 items (34.1%) obtained a CVC between 0.70 and 0.79, which indicates they were valid but open to revision or improvement, particularly regarding wording or placement within the structure of the guide. Finally, seven items (15.9%) presented a CVC < 0.70, for which restructuring or elimination was recommended after further qualitative review.

Table 2 summarizes the results for both Aiken's V and the corrected CVC values for each item of the interview guide. The table also provides an integrated interpretation: items are considered valid when both coefficients meet or exceed the 0.80 threshold; revisable when at least one coefficient falls between 0.70 and 0.79; and requiring priority revision when one or both fall below 0.70, or when qualitative feedback highlighted concerns. This classification guided subsequent decisions to modify, reformulate, or reposition items in the final validated version of the instrument.

**Table 2:** Results of Aiken's V and corrected CVC for each item of the interview guide.

Item	Aiken's V	Corrected CVC	Final interpretation
1.1	1.00	0.96	Valid
1.2	1.00	0.96	Valid
1.3	0.95	0.80	Revisable
1.4	1.00	0.96	Valid
1.5	0.90	0.76	Priority revision
1.6	1.00	0.96	Valid
1.7	1.00	0.96	Valid
1.8	1.00	0.96	Valid
2.1	0.95	0.78	Revisable
2.2	1.00	0.88	Valid
2.3	0.75	0.64	Priority revision
2.4	0.80	0.68	Priority revision
3.1	0.75	0.64	Priority revision
3.2	0.85	0.72	Revisable
3.3	0.90	0.76	Revisable
3.4	0.75	0.64	Priority revision
3.5	0.90	0.76	Revisable
3.6	0.75	0.64	Priority revision
4.1	0.95	0.84	Valid
4.2	1.00	0.92	Valid
4.3	1.00	0.92	Valid
4.4	0.90	0.76	Revisable
5.1	0.85	0.72	Priority revision
5.2	0.95	0.88	Valid
5.3	0.95	0.88	Valid
5.4	0.90	0.76	Revisable
5.5	0.95	0.88	Valid
6.1	1.00	0.96	Valid
6.2	0.85	0.72	Revisable
6.3	0.90	0.76	Revisable
6.4	1.00	0.96	Valid
7.1	0.90	0.76	Revisable
7.2	0.90	0.76	Revisable
7.3	0.90	0.76	Revisable
7.4	0.75	0.64	Priority revision
7.5	0.85	0.72	Revisable
8.1	0.80	0.68	Priority revision
8.2	0.90	0.76	Revisable
8.3	0.95	0.88	Valid
9.1	0.90	0.76	Revisable
9.2	0.85	0.72	Revisable
9.3	0.70	0.60	Priority revision
10.1	0.95	0.88	Valid
10.2	1.00	0.96	Valid

Source: own elaboration.

### 4.2 Qualitative results

The qualitative analysis of the experts’ feedback complemented and enriched the quantitative findings, adding substantial value to the validation process. Overall, the qualitative assessments were positive, highlighting the suitability of the guide to the study's objectives and the sociocultural characteristics of the target population. Nevertheless, the experts provided a series of critical suggestions aimed at improving key aspects of the instrument, including wording, logical order of sections, cultural sensitivity, and methodological clarity.

The qualitative contributions helped refine the instrument in terms of accessibility, contextual relevance, and semantic precision. The most relevant recommendations included:

- Reordering sections to enhance the narrative flow and gradual construction of the participants’ stories (e.g., moving sociodemographic data to the end of the interview).
- Adding missing variables, such as family situation (marital status, children in care), to the sociodemographic section.
- Reformulating items with overly technical or ambiguous wording, replacing terms potentially inaccessible for women in vulnerable social situations (e.g., replacing “limitations” or “urban laboratories” with more understandable and culturally sensitive expressions such as “influences” or “meeting places”).
- Splitting or simplifying lengthy items or those containing multiple subquestions, in order to avoid confusion, reduce cognitive load, and facilitate comprehension (e.g., items 3.4 and 4.4).
- Eliminating or rephrasing items deemed overly complex or unfeasible from a methodological or ethical standpoint, such as item 7.4.
- Introducing concrete examples and participatory formats for items exploring community engagement, to help participants identify with the proposed content (e.g., item 9.3).

Item 9.3 received the lowest scores in both Aiken's V and corrected CVC. Experts agreed it was excessively technical and recommended a reformulation with more accessible examples and a clearer, more direct structure.

The research team also decided to retain certain items considered conceptually essential - for instance, those addressing experiences of discrimination, gender, or public policies - even if they received intermediate scores in the quantitative analyses. These items were reformulated with greater semantic sensitivity to ensure comprehension while preserving their critical content.

As a result of the review and improvement process, the interview guide was expanded from 44 to 54 items. This increase reflects the added value of the mixed-methods approach and the richness of qualitative feedback, leading to a more robust, contextualized, and culturally sensitive instrument.

 Table 3 systematizes the most relevant qualitative contributions from each expert.

**Table 3:** Main qualitative contributions from experts in the validation of the interview guide.

Expert	Main qualitative contributions
1	- Replace “time of residence” with “time living in Spain.” - Clarify that physical activity refers to “leisure time.” - Add “/exclusive” after “inclusive.” - Replace “coexistence between cultures” with “approach between cultures.” - Eliminate the term “urban laboratory.”
2	- Reorder sections: move sociodemographic data to the end; begin with migration background. - Replace terms such as “perspective” with “point of view,” or “barriers” with “difficulties.” - Reformulate complex or sensitive questions (e.g., item 7.4, item 1.5). - Standardize writing style (verb tense, clarity, syntax). - Propose more neutral wording and specific questions (frequency, location, with whom). - Recommend eliminating technical terms such as “urban laboratory” and replacing them with alternatives like “meeting spaces.”
3	- Replace “limit” with “influence” in items on social and gender norms. - Suggest adding a question on experiences of discrimination (moving item 5.3 to a new section as 2.5). - Use more accessible wording instead of long questions. - Add questions related to the Spanish cultural context and its inclusiveness. - Propose examples and new participatory questions (e.g., item 9.4). - Include aspects of mental health, type of social support, and gender differences.
4	- Add the variable “family situation” to sociodemographic data. - Differentiate between regulatory and social norms. - Include subquestions on safety, transportation, language, and perception of space. - Incorporate indicators of social participation (where they practice, with whom, frequency). - Add details on public policies and the role of associations. - Recommend clarifying the design and implementation of rules: distinguish between administration and organizations.
5	- Postpone sensitive questions such as reasons for migration (item 1.5). - Divide long items or those with multiple implicit questions (e.g., 3.4, 9.3). - Add the variable of mental health when assessing the impact of physical activity. - Avoid biased terms such as “negative”; propose neutral alternatives. - Consider language as a significant potential barrier. - Reconsider overly technical or difficult-to-understand items for newcomers. - Introduce a comparative cultural perspective in questions on gender and participation.

Source: own elaboration.

These contributions informed the final version of the interview guide, which incorporated adjustments in wording and structure as well as conceptual refinements, based on consensus between quantitative and qualitative analyses.

The final instrument (Supplementary Appendix) consists of 54 items distributed across ten thematic sections addressing key dimensions related to physical activity, social inclusion, and acculturation processes among immigrant women. The questions were reformulated with sensitivity, clarity, and cultural appropriateness, with the goal of generating an accessible and respectful dialogue that enables the collection of meaningful and in-depth qualitative data.

## 5 Discussion

Although the interview guide developed in this study constitutes a pioneering tool specifically designed to explore the role of physical activity in urban public spaces in the social inclusion of immigrant women, the validation process applied is firmly grounded in established methodological practices within sport, physical activity, and social research. Previous studies have successfully validated qualitative and mixed-method instruments through expert judgment combined with quantitative indices such as Aiken's V coefficient and the CVC, supporting their suitability for assessing content validity (21, 22, 24, 28). In this sense, the approach adopted in the present study aligns with recognized validation strategies while responding to the need for instruments specifically tailored to the experiences of immigrant women in urban contexts.

From a methodological standpoint, the integration of expert judgment with psychometric techniques—specifically Aiken's V coefficient (26) and the CVC (20)—provided a solid and transparent basis for assessing the relevance and clarity of the interview items. The high degree of agreement observed among experts (global Aiken's V = 0.89; global CVC = 0.88) confirms the overall adequacy of the instrument, while simultaneously allowing for the identification of items requiring refinement. This mixed-method validation strategy, combining quantitative indicators with qualitative expert feedback, is consistent with current methodological recommendations advocating complementary approaches in the assessment of qualitative research instruments (21).

In the specific field of physical activity and sport sciences, the application of the CVC remains limited but methodologically significant. One of the few validation studies explicitly using the CVC within this domain is the work by Pérez-Morales et al. (29), who employed the coefficient to assess the content validity of an instrument designed to evaluate tactical procedural knowledge in basketball. Their study demonstrated that the CVC is a suitable and rigorous method for ensuring the clarity and practical relevance of items in sport-specific assessment tools.

The relative scarcity of studies applying the CVC in physical activity and sport research does not undermine its methodological value; rather, it highlights the need for further applications that extend its use beyond performance-oriented instruments toward social, educational, and community-based contexts. In this sense, the present study contributes to the advancement of validation practices in the field by applying the CVC to a qualitative interview guide focused on physical activity in urban public spaces and social inclusion, an area that has received limited methodological attention to date.

Beyond methodological considerations, the relevance of the interview guide also lies in its theoretical positioning. It adopts an intersectional framework that enables a nuanced understanding of immigrant women's experiences by considering the interlocking effects of gender, ethnicity, class, and migration status (2, 12). Unlike previous studies that have addressed these dimensions in isolation, the present instrument integrates access to public and sports spaces, perceptions of safety, cultural norms, discrimination, and community participation within a single analytical framework. This integrative approach represents a methodological advance consistent with prior critical work in sport and migration studies (3, 4, 11).

By explicitly situating physical activity within urban public spaces, the guide operationalizes key assumptions of feminist and critical urban scholarship. Physical activity is conceptualized not merely as an individual health-related behavior, but as a socially embedded practice through which immigrant women negotiate visibility, belonging, and legitimacy in the urban environment. In this sense, access to and appropriation of public space emerge as central mechanisms through which processes of inclusion and exclusion are produced, reproduced, and contested. This conceptualization strengthens the analytical potential of the instrument and situates it firmly within contemporary debates on urban citizenship, gender, and social justice.

An additional strength of the study lies in the potential development of a parallel version of the interview guide for immigrant men. Such an extension would enable comparative gender analyses from a relational perspective, contributing to a more comprehensive understanding of how gender shapes experiences of migration, physical activity, and the appropriation of urban public spaces (18, 30). This comparative potential enhances the analytical scope of the instrument and opens new avenues for future research on gendered dynamics in migration contexts.

Several limitations should nonetheless be acknowledged. First, although the expert validation process ensured a rigorous assessment of content validity, the absence of a pilot study limits the evaluation of the guide's performance in real interview settings. While this decision was methodologically justified, future applications could benefit from pilot testing to further assess comprehension, narrative flow, and contextual adaptability (31, 34). Second, although the number of experts involved (*n* = 5) meets established methodological standards for content validation studies (19, 21), expanding the expert panel in future validations could enhance disciplinary, cultural, and linguistic diversity, thereby strengthening the robustness of the validation process.

Importantly, the methodological design is explicitly grounded in constructivist grounded theory, which conceives research instruments as flexible and evolving rather than fixed and closed (9, 32). In this sense, the interview guide is designed to be progressively refined through empirical application. As interviews are conducted, emerging insights are expected to allow for the adjustment and enrichment of items, functioning as an ongoing internal validation process embedded within the fieldwork itself.

Finally, while the guide was developed within the Spanish context—where specific urban, institutional, and policy-related factors shape immigrant women's experiences—many of its thematic axes are transferable to other sociocultural settings. With appropriate contextual adaptations, the instrument may be applied in different national contexts or with other migrant populations, thereby enhancing its comparative and international relevance.

Overall, the validated interview guide not only meets high technical standards but also constitutes an epistemologically coherent tool aligned with feminist, critical, and intersectional research principles (9, 10). Instruments of this nature are essential for producing situated, ethical knowledge capable of informing more inclusive public policies and interventions in the domains of physical activity, urban planning, health, and social inclusion.

## 6 Conclusion

This study validated a qualitative interview guide designed to explore processes of social inclusion and acculturation among immigrant women through physical activity in urban public spaces. The methodological strategy employed - based on expert judgment and the combined use of psychometric and qualitative techniques - enabled the development of a clear, relevant, and socially contextualized instrument.

The resulting guide constitutes a methodological tool for collecting qualitative information on the multiple dimensions that shape the migrant experience in urban environments, including sociocultural, institutional, and symbolic aspects. Its intersectional orientation and flexible structure make it particularly well suited for research addressing complex social phenomena at the intersection of gender, migration, and urban life.

Based on these results, the following research avenues are proposed:

1. Empirical application of the guide in interviews with immigrant women from diverse backgrounds and urban settings.
2. Comparative gender studies using adapted versions of the instrument designed for immigrant men.
3. Longitudinal analyses exploring changes in the use of public space and support networks over time.
4. Cross-cultural validations of the guide in other national or international contexts.

In conclusion, the validation presented here provides a methodologically robust and socially relevant instrument for investigating how physical activity in urban spaces can act as a vehicle for social inclusion. Its careful design and intersectional approach make it a significant contribution for future studies in the fields of public health, sociology of sport, and urban anthropology, as well as for informing more inclusive and equitable social interventions.

## Data Availability

The original contributions presented in the study are included in the article/Supplementary Material, further inquiries can be directed to the corresponding author.
