# Supplementary material for: Design and validation of a qualitative interview for the study of the role of physical activity in urban public spaces in the social inclusion of immigrant women (Spanish version)
Source: Front Sports Act Living. 2026 Mar 2;8:1717266. doi: 10.3389/fspor.2026.1717266 (PMC12989535; doi:10.3389/fspor.2026.1717266)
Supplement: Supplementary file 1 [file Datasheet1.pdf]

APPENDIX: VALIDATED INTERVIEW (SPANISH VERSION)

SECCIÓN 1. Antecedentes y condicionantes migratorios

Objetivo: Explorar el contexto previo y condicionantes culturales que influyen en la práctica de actividad física

1.1. ¿En el país donde tú vivías, qué significado tenía para ti la práctica de actividad física o deporte? (ocio, salud, convivencia, obligación, tradición, religión…)
1.2. ¿Ha cambiado este significado desde que migraste?
1.3. ¿Había influencias sociales o familiares (normas sociales, reglas escritas, valores, creencias) que afectaran tu acceso a la actividad física o deportiva?
1.4. ¿Practicabas sola o con otras personas (mujeres, grupos mixtos)? ¿Cómo era esa experiencia?
1.5. ¿Qué tipo de actividades físicas practicabas en tu país de origen y
1.6. ¿Con qué frecuencia?
1.7. ¿Experimentaste alguna vez discriminación o incomodidad al realizar actividad física en tu país de origen? ¿Cómo fue esa experiencia?

SECCIÓN 2. Experiencia actual con la actividad física en el tiempo libre y espacios deportivos

Objetivo: Evaluar la participación actual y los factores que la favorecen o limitan

2.1. ¿Has encontrado dificultades o ayudas al intentar mantener hábitos de actividad física durante el proceso migratorio y al llegar a España?
2.2. Al llegar a España, ¿qué factores o motivos influyeron en tu decisión de practicar (o no) actividad física o deporte?
2.3. ¿Qué tipo de actividad física o deporte practicas en tu tiempo libre actualmente?
2.4. ¿Cómo y dónde realizas estas actividades? ¿Con quién las practicas?
2.5. ¿Con qué frecuencia practicas? ¿Consideras que es suficiente? ¿Por qué?
2.6. ¿Te gustaría cambiar algo de tu práctica de actividad física o deportiva? ¿El qué?
2.7. Si no participas actualmente o no tanto como te gustaría, ¿qué te impide hacerlo? ¿Podrías explicar cómo estas dificultades afectan tu participación?
☐ Barreras económicas ☐ Responsabilidades familiares ☐ Miedo a la discriminación ☐ Falta de tiempo ☐ Desconocimiento de espacios ☐ Problemas de salud ☐ Percepción de seguridad ☐ Acceso/transporte ☐ Idioma ☐ Otro (especificar): ________

SECCIÓN 3. Uso y percepción de espacios públicos

Objetivo: Explorar el acceso físico y simbólico a los espacios deportivos urbanos

3.1. ¿Utilizas espacios públicos (parques, pistas, plazas, etc.) para hacer actividad física? ¿Cuáles?
3.2. ¿Cómo conociste esos espacios (amistades, redes sociales, asociaciones…)?
3.3. ¿Qué valoración haces sobre estos espacios en cuanto a accesibilidad, seguridad, comodidad, ambiente…?
3.4. ¿Crees que estos espacios son inclusivos o excluyentes con mujeres inmigrantes? ¿Por qué?
3.5. ¿Podrías compartir alguna experiencia positiva o negativa en estos espacios?

SECCIÓN 4. Inclusión social y redes de apoyo

Objetivo: Analizar cómo las redes comunitarias inciden en la inclusión social

4.1. ¿Has encontrado apoyo o ayuda en asociaciones, redes comunitarias o vecinales desde que estás en España?

4.2. ¿Has conocido nuevas personas a través de la actividad física? ¿Cómo han influido estas relaciones en tu vida?
4.3. ¿Te has sentido discriminada o incómoda al practicar actividad física con otras personas en España? ¿Qué sentiste o hiciste?
4.4. ¿Has recibido información o apoyo específico para acceder a actividades físicas o deportivas desde alguna entidad o colectivo?
4.5. ¿Tienes personas cercanas (familia, pareja, amistades) que te apoyen en tu práctica de actividad física o deporte?

SECCIÓN 5. Bienestar personal y salud
Objetivo: Explorar el impacto emocional y psicológico de la actividad física
5.1. ¿Cómo te hace sentir la práctica de actividad física a nivel físico, emocional y mental?
5.2. ¿Consideras que la actividad física ayuda a mejorar las relaciones sociales entre personas de distintos orígenes y culturas? ¿Por qué?
5.3. ¿Desde que practicas actividad física, ha cambiado tu forma de relacionarte con personas españolas o con la cultura local y viceversa? ¿De qué manera?
5.4. ¿Sientes que la actividad física te ayuda a sentirte parte del lugar donde vives?

SECCIÓN 6. Percepciones de género en la actividad física
Objetivo: Analizar diferencias y dificultades específicas según el género
6.1. ¿Existen diferencias entre cómo hombres y mujeres practican actividad física en tu país de origen? ¿Y en tu comunidad actual? ¿Cómo son esas diferencias?
6.2. ¿Has tenido más dificultades o facilidades para acceder o participar en actividades físicas por ser mujer? ¿Cuáles?
6.3. ¿Has vivido alguna situación en la que el hecho de ser mujer haya afectado tu experiencia en la práctica de actividad física (comentarios, actitudes, reglas implícitas…)?
6.4. ¿Cómo es la experiencia de participar en actividad física o deportiva con otras mujeres?
6.5. ¿Qué opinas sobre que hombres y mujeres compartan espacios y actividades físicas? ¿Te parece positivo, negativo o depende?

SECCIÓN 7. Políticas públicas y participación
Objetivo: Relacionar la experiencia individual con la acción institucional
7.1. ¿Conoces las diferencias entre el papel de la administración pública y el papel de las asociaciones en el acceso tu participación en actividad física o deporte?
7.2. ¿Consideras que la administración pública o las asociaciones tienen un papel facilitador o limitante en tu acceso a la práctica deportiva? ¿Por qué?
7.3. ¿Consideras importante que desde las administraciones públicas se desarrollen programas de actividad física dirigidos a mujeres inmigrantes?
7.4. ¿Qué cambios harías tú, desde tu punto de vista, para mejorar los espacios deportivos públicos y hacerlos más accesibles a mujeres inmigrantes?
7.5. ¿Has participado en algún programa público o comunitario relacionado con la actividad física? ¿Cómo fue tu experiencia?

SECCIÓN 8. Espacios de encuentro participativo
Objetivo: Recoger propuestas y disposición para iniciativas comunitarias
8.1. ¿Te gustaría participar en encuentros grupales con otras personas para hablar sobre la actividad física y la vida cotidiana en este país?

8.2. ¿Qué temas relacionados con la actividad física o el uso de espacios públicos te gustaría tratar en estos encuentros?
8.3. ¿Te gustaría participar en actividades grupales? ¿Qué tipos te parecen más interesante? (puedes marcar varios)
☐ Actividades físicas comunitarias y accesibles ☐ Dinámicas de diálogo y participación ☐ Actividades intergeneracionales e interculturales ☐ Microproyectos de transformación urbana ☐ Actividades formativas y de sensibilización ☐ Otras (especificar): __________
8.4. ¿Tienes alguna sugerencia para crear otras formas, oportunidades o espacios donde las personas puedan encontrarse y participar activamente?

SECCIÓN 9. Datos sociodemográficos
Objetivo: Contextualizar las respuestas
9.1. Edad: _____
9.2. País de origen: _____
9.3. Tiempo viviendo en España: _____
9.4. Ciudad/población actual: _____
9.5. Nivel educativo: Sin estudios / Primaria / Secundaria / Universitario / Otro (especificar): _____
9.6. Situación laboral y/o educativa actual: _____
9.7. Nivel de conocimiento del idioma español (escala del 1 al 5): _____
9.8. Situación familiar: ☐ Soltera ☐ En pareja ☐ Hijos/as a su cargo ☐ Otro (especificar): __________
9.9. Motivo principal por el que migraste: __________

SECCIÓN 10. Reflexiones finales y cierre
Objetivo: Recoger observaciones adicionales que puedan enriquecer el análisis cualitativo
10.1. Imagina que una mujer acaba de llegar a España y quiere empezar a hacer actividad física pero no sabe cómo. ¿Qué consejo le darías? ¿Por qué crees que es importante comenzar cuanto antes?
10.2. ¿Hay algo más que te gustaría compartir sobre tu experiencia personal con la actividad física, la inclusión social o tu vida cotidiana en este país?
